# *MDM4* genetic variants predict HPV16-positive tumors of patients with squamous cell carcinoma of the oropharynx

**DOI:** 10.18632/oncotarget.21414

**Published:** 2017-09-30

**Authors:** Zhongming Lu, Hua Zhang, Ye Tao, Xiangping Li, Guojun Li

**Affiliations:** ^1^ Department of Otolaryngology-Head and Neck Surgery, Nanfang Hospital, Southern Medical University, Guangzhou, Guangdong 510515, China; ^2^ Department of Head and Neck Surgery, The University of Texas MD Anderson Cancer Center, Houston, TX 77030, USA; ^3^ Department of Otolaryngology-Head and Neck Surgery, Guangdong General Hospital and Guangdong Academy of Medical Sciences, Guangzhou, Guangdong 510080, China; ^4^ Department of Otolaryngology-Head and Neck Surgery, The Affiliated Yantai Yuhuangding Hospital of Qingdao University, Yantai, Shandong 264000, China; ^5^ Department of Otolaryngology-Head and Neck Surgery, The 2nd Affiliated Hospital of Anhui Medical University, Hefei, Anhui 230601, China; ^6^ Department of Epidemiology, The University of Texas MD Anderson Cancer Center, Houston, TX 77030, USA

**Keywords:** *MDM4* polymorphism, genetic susceptibility, HPV, SCCOP, biomarkers

## Abstract

The increasing incidence of squamous cell carcinoma of the oropharynx (SCCOP) is majorly attributed to the human papillomavirus (HPV) infection. Both HPV and MDM4 play a critical role in inhibition of p53 activity, thus affecting HPV tumor status of SCCOP. Three polymorphisms in *MDM4* were genotyped from blood genomic DNA samples and HPV16 status in tumor specimens was examined. Odds ratio (OR) and 95% confidence intervals (CIs) in univariate and multivariable logistic regression models were calculated for the associations between these polymorphisms and HPV16 status. Three *MDM4* variant genotypes were significantly associated with HPV16 tumor status among SCCOP patients compared with the common homozygous genotypes (OR, 0.6; 95% CI, 0.4–1.0 for rs10900598; OR, 1.6, 95% CI; 1.1–2.4 for rs1380576; and OR, 1.8, 95% CI, 1.1–2.9 for rs11801299; respectively). When we combined all risk genotypes of the 3 polymorphisms, the patients carrying 1-3 *MDM4* risk genotypes were approximately 2.5 time as likely to have an HPV16-positive tumor than those with no risk genotypes (OR, 2.5, 95% CI, 1.6–3.9). Additionally, modifying effect of *MDM4* risk genotypes was more pronounced among non-Hispanic white, never-smokers, and never-drinkers. Potential functional polymorphisms in *MDM4* may serve as biomarkers for predicting tumor HPV16 status among SCCOP patients, particularly in non-Hispanic white, never-smokers and never-drinkers. However, validation of these results in larger studies is needed.

## INTRODUCTION

Historically, the majority of SCCHN are contributed to tobacco and alcohol use. However, Squamous cell carcinoma of the oropharynx (SCCOP), one of subgroups of SCC of head and neck (SCCHN), has recently become one of the only five cancer types that are growing significantly in incidence regardless of the decline in smoking rate in the United States [1]. Human papillomavirus (HPV) infection has been well established as the principal cause for the increased incidence of SCCOP [1–7]. Among over 150 known HPV subtypes, the HPV 16 is the most common subtype for SCCOP and accounts for up to 95% of HPV-positive cases [8]. HPV16-positive SCCOP has been widely recognized to be a distinct disease and has better treatment outcome compared with HPV16-negative SCCOP [9, 10]. Besides HPV infection, as a necessary but not a sufficient cause of SCCOP, other factors, such as genetic factors, may also be necessary in malignant transformation of HPV infected cells.

*Mouse double minute 4* (*MDM4* or *MDMX*) is a homolog of MDM2, a key gene for negatively regulating p53, and its expression alterations may contribute to cancer development through p53 inhibition. p53 has been regarded as “the guardian of the genome” [11] and genetic alterations in the p53-dependent pathway play a crucial role in regulating expression of various genes in many cancers including SCCOP [12]. It has been revealed that MDM4 exerts its oncogenic activity by binding to the p53 transactivation domain, leading to inhibition of its transcriptional activity along with MDM2 oncoprotein [13]. Previous study suggested that functional single nucleotide polymorphisms (SNPs) of these p53-related genes, including *MDM4* and *MDM2*, could predict the risk of HPV-associated SCCHN [14]. HPV E6 protein, a major HPV oncogenic protein [15], activates telomerase and binds p53 for degradation via the ubiquitination, leading to protein dysfunction, degradation, and loss of cell cycle control through an ubiquitin-dependent pathway [16]. However, only a very small proportion of HPV-infected individuals ultimately develop HPV-positive SCCOP [17], indicating that other genetic variations may also be attributed to HPV tumor status of SCCOP.

Given the distinct roles of HPV E6 and MDM4 in regulation of p53, with a direct degradation of p53 by HPVE6 and an inhibition of p53 transcription by MDM4, both leading to downexpression of p53, the possible link in mechanisms on p53 downregulation by HPV E6 and MDM4 is less investigated. The degradation of Rb by E7 releases the E2F transcription factors, resulting in the uncontrolled cell cycle and cell proliferation [18]. The released E2F will activate the p14 transcription and lead to the dissociation of p53 from MDM2, a homolog of MDM4, inducing p53-dependent apoptosis [18]. Thus, MDM4 may have a similar effect on p53 activity through its link with HPV, while this hypothesis warrants further future investigation. Some studies have indicated the development of SCCOP associated with HPV16 may be affected by *MDM4* genetic variants [18], while whether *MDM4* variants are associated with tumor HPV16 status in SCCOP patients remains unknown. Thus, given the role of HPV16 status as a biomarker for predicting prognosis of SCCOP, we evaluated whether *MDM4* polymorphisms could be served as a susceptibility biomarker for HPV16 tumor status in SCCOP patients.

## RESULTS

The distribution of 552 SCCOP patients’ demographic characteristics as well as smoking and alcohol history is summarized in Table 1. Among these 552 patients with SCCOP, 439 (79.5%) were positive and 113 (20.5%) were negative for tumor HPV16 DNA. HPV16-positive patients have higher possibility of man and never-smokers than HPV16-negative patients (*P* = 0.0012 for sex and *P* = 0.050 for smoking status, respectively). Additionally they were more likely to be non-Hispanic white and never drinkers, but the statistical difference were not significantly different (*P* = 0.141 for race and *P* = 0.483 for alcohol status, respectively).

**Table 1:** Demographic characteristics of SCCOP patients by HPV16 status

Variable	HPV16^+^ patients (N =439)		HPV16^-^ patients (N =113)		*P* value*
	No.	%	No.	%	
Age					0.856
≤54 years	225	51.2	59	52.2	
>54 years	214	48.8	54	47.8	
Sex					0.0012
Male	384	87.5	85	75.2	
Female	55	12.5	28	24.8	
Ethnicity					0.141
Non-Hispanic white	404	92.0	99	87.6	
Others	35	8.0	14	12.4	
Tobacco smoking					0.050
Ever	252	57.4	76	67.0	
Never	187	42.6	37	33.0	
Alcohol drinking					0.483
Ever	331	75.4	88	78.6	
Never	108	24.6	25	21.4	

*Two-sided χ^2^ test.

Table 2 shows the genotype distribution of these 3 *MDM4* polymorphisms, rs10900598, rs1380576, and rs11801299. It is less likely to have the *MDM4* rs10900598 polymorphism variant GT/TT genotypes for HPV16-positive patients (67.2%) than HPV16-negative patients (77.0%; *P* = 0.044), while HPV16-positive subjects more likely had the rs1380576 CG+GG genotypes than HPV16-negative subjects (57.6% and 45.1%, respectively; *P* = 0.017). Similarly, for the rs11801299 polymorphism, the AG+AA variant genotypes were more likely in HPV16-positive patients (37.6%) than in HPV16-negative patients (25.6%; *P* = 0.018). Such a significant difference was also observed when genotypes were combined; HPV16-positive patients more likely had risk genotypes than HPV16-negative patients (80.6% vs. 61.9%, respectively; *P* = 0.003) (Table 2).

**Table 2:** Risk of SCCOP associated with the *MDM4* genotypes in HPV16 ^+^ and HPV16^-^ patients

*MDM4* genotypes	HPV16^+^ patients (N =439)		HPV16^-^ patients (N =113)		*P*	Crude OR (95% CI)	Adjusted OR (95% CI)*
	No.	%	No.	%			
rs10900598					0.044		
GG^†^	144	32.8	26	23.0		1.0	1.0
GT + TT	295	67.2	87	77.0		0.6 (0.4-1.0)	0.6 (0.4-1.0)
rs1380576					0.017		
CC^†^	186	42.4	62	54.9		1.0	1.0
CG + GG	253	57.6	51	45.1		1.7 (1.1-2.5)	1.6 (1.1-2.4)
rs11801299					0.018		
GG^†^	274	62.4	84	74.4		1.0	1.0
AG + AA	165	37.6	29	25.6		1.7 (1.1-2.8)	1.8 (1.1-2.9)
Combined risk genotypes					0.003		
Risk 0	85	19.4	43	38.1		1.0	1.0
Risk 1-3	354	80.6	70	61.9		2.6 (1.6-4.0)	2.5 (1.6-3.9)

P values for χ^2^ test for genotype distribution.

*Adjusted for age, sex, ethnicity, tobacco smoking and alcohol drinking status in a logistic regression model.

^†^Reference group

*MDM4* rs10900598 GT/TT, rs1380576 CC, and rs11801299 GG were considered as risk genotypes.

In multivariable analyses after adjusting with age, sex, ethnicity, smoking and alcohol status, a significant difference was noticed for all three polymorphisms. The patients carrying the variant genotypes of *MDM4*rs10900598 had 60% lower risk to have an HPV16-positive tumor than those with the corresponding homozygous genotype (OR, 0.6, 95%CI, 0.4-1.0), while the patients with variant genotypes of *MDM4*rs1380576 (CG+GG) and *MDM4*rs11801299 (AG+AA) were approximately 1.6 and 1.8 folds as likely to be HPV16-positive tumors compared to the cases with their corresponding homozygous genotypes (OR, 1.6, 95%CI, 1.1-2.4 for *MDM4*rs1380576 and OR, 1.8, 95%CI, 1.1-2.9 for *MDM4*rs11801299). Furthermore, after we combined the risk genotypes of the 3 polymorphisms, HPV16-positive SCCOP patients were 2.5 times more likely to have any risk genotypes of the 3 polymorphisms compared with HPV16-negative patients (OR, 2.5, 95% CI, 1.6-3.9) (Table 2).

As shown in Table 3, we further performed stratified analyses by age, sex, ethnicity, and tobacco smoking and alcohol drinking status to further explore the association between the combined genotypes of three *MDM4* polymorphisms and tumor HPV16 status of SCCOP (Table 3). Among patients under 55 years old, those with any risk genotypes of the 3 polymorphisms were 1.6 times more likely to have a HPV16-positive tumor than those without any variant genotypes (OR, 1.6; 95% CI, 1.0-3.0); however, no significant association was found for patients older than 54 years. Additionally, patients with any risk genotypes of the 3 polymorphisms were more likely to be HPV16-positive SCCOP among non-Hispanic whites (OR, 2.3; 95% CI, 1.4-3.8), and those who were never smokers (OR, 2.3; 95% CI, 1.1-5.0) as well as never alcohol drinkers (OR, 4.6; 95% CI, 1.8-12.2).

**Table 3:** Stratification analysis of *MDM4* genotypes, OR, and 95% CIs by selected variables

Variable	HPV16^+^ patients (N =439)				HPV16^-^ patients (N =113)				Adjusted OR (95%CI)^*^	
	No risk		With 1-3 risk		No risk		With 1-3 risk		No variants^†^	With variants
	No.	%	No.	%	No.	%	No.	%		
All subjects	85	19.4	354	80.6	43	38.1	70	61.9	1.0	2.5 (1.6-3.9)
Age										
≤54 years	50	22.2	175	77.8	18	30.5	41	69.5	1.0	1.6 (1.0-3.0)
>54 years	35	16.4	179	83.6	25	46.3	29	53.7	1.0	1.9 (0.9-2.7)
Sex										
Male	75	19.5	309	80.5	32	37.6	53	62.4	1.0	2.8 (1.4-3.9)
Female	10	18.2	45	81.8	11	39.3	17	60.7	1.0	3.1(1.1-9.2)
Ethnicity										
Non-Hispanic White	79	19.6	325	80.5	37	37.4	62	62.6	1.0	2.3 (1.4-3.8)
Others	6	17.1	29	82.9	6	42.9	8	57.1	1.0	4.6 (0.9-21.7)
Smoking status										
Ever	51	20.2	201	79.8	29	38.7	46	61.3	1.0	1.6 (0.7-3.5)
Never	34	18.2	153	81.8	13	35.1	24	64.9	1.0	2.3 (1.1-5.0)
Drinking status										
Ever	66	19.9	265	80.1	30	34.1	58	65.9	1.0	2.0 (0.9-4.0)
Never	19	17.6	89	82.4	12	50.0	12	50.0	1.0	4.6 (1.8-12.2)

*Adjusted for age, sex, ethnicity, tobacco smoking and alcohol drinking status in a logistic regression model.

^†^Reference group.

## DISCUSSION

In the present study, a significant association between the three *MDM4* polymorphisms and HPV16 tumor status in patients with SCCOP were found; and such associations were more pronounced among some subgroups. This result indicates that these *MDM4* genetic variants might have potential to be biomarkers for HPV16-posoitive tumors of SCCOP, particularly in the patients who were younger, non-smokers, and non-drinkers. To date, this is the first study to explore the association of *MDM4* polymorphisms with tumor HPV16 status in SCCOP.

Identifying reliable predictors of HPV status in SCCOP is clinically useful since tumor HPV status remains the strongest marker for predicting outcome of SCCOP. Our results indicate that the combination of variant genotypes of the 3 *MDM4* variants may be use to serve as more valuable markers for the HPV tumor status of SCCOP. Understanding the tumor HPV16 status of SCCOP may help make treatment decision. Since HPV-positive SCCOP patients have better treatment response and prognoses than HPV-negative SCCOP patients [19], it is likely to help reduce the treatment intensity currently used for HPV-positive SCCOP patients as well as to develop future targeted therapies for such patients. The results from *MDM4* genetic variants could define personalized molecular profiling for future personalized prevention and potentially optimizing patient stratification for HPV16-targeted therapies, leading to better individualized treatment and improved prognosis of SCCOP patients.

Our study demonstrated that some genetic variants of *MDM4* may individually or jointly modify tumor HPV16 status in SCCOP. Although some studies have reported associations of some *MDM4* genetic variants with HPV-associated head and neck cancers [20–24], these studies either defined HPV16 status of study patients by serology or had mixed tumor sites [20, 21, 23, 24]. Although these studies imply that HPV tumor positivity might have favorable prognosis of SCCOP, these findings are not in agreement with those from other studies [25–27]. It is likely that some other important factors may also significantly confound the estimates of the associations. Therefore, to ensure future treatment of SCCOP, HPV tumor positivity as a potential biomarker for prognosis requires further validation. The findings from this study indicate that *MDM4* polymorphisms might serve as a biomarker for the HPV16-positive tumors of SCCOP. Since this study included tumor-based HPV16 status and a homogenous subgroup of SCCOP, it might minimize the selection bias on the associations of *MDM4* polymorphisms with tumor HPV16 status of SCCOP.

While how these polymorphisms exactly affect the tumor HPV16 status of SCCOP remains unclear, it is biologically plausible that HPV16 E6 and MDM4 may jointly affect development of SCCOP by degrading or inhibiting p53. It is biologically that HPV E6 inhibits p53 through proteasomal degradation [28], and MDM4 directly binds to the p53 transactivation domain for inhibition of p53. Thus, the overexpression of MDM4 could inhibit the p53 activity, and functionally affect p53-dependent pathway, thus affecting several p53-related cellular activities. This altered regulation may affect the interaction of p53 with HPV16 through degradation or inactivation of HPV16 E6 oncoprotein [16], thus could suppressing cell growth and inducing apoptosis. It is likely that *MDM4* variants could jointly modify association with tumor HPV16 status in SCCOP by interaction with HPV16 E6 and p53*.*

It is well known that patients with HPV16-positive SCCOP tended to be likely non-smokers or non-drinkers. Indeed, our findings from stratification analysis have demonstrated that modifying effect of combined *MDM4* risk genotypes on SCCOP tumor HPV16 positivity was much higher in never-smokers and never-drinkers. Such a finding could additionally support those results from previously reported work. In these studies, they demonstrated that HPV infection may cause a significant portion of SCCOP, whereas smoking and drinking induced the majority of non-SCCOP [29, 30]. Since HPVs can bypass immune systems, and *MDM4* polymorphisms can modulate the apoptotic capacity though p53 pathway, both molecular pathways eventually controlling the HPV clearance through escape of immune surveillance, subsequently affecting the tumor HPV status [31]. Additionally, the association of tumor HPV16 status with combined *MDM4* risk genotypes in SCCOP was more evident among ever-drinkers in the current study, implying HPV16 could interact with alcohol and smoking, even though non-drinkers more likely had HPV-positive tumors than ever-drinkers [32]. Furthermore, smokers or alcohol drinkers may synergize with *MDM4* polymorphisms to affect HPV16 status through suppression of immune systems, clearing the HPV-infected cells and thus less likely to be tumor HPV16-positive SCCOP. Nevertheless, these findings need to be further validated in future studies with large sample sizes.

In interpretation of our findings some limitations should be taken into account. Several strengths from this study are: 1) inclusion of single SCCOP tumor site only; 2) use of HPV16 tumor status rather than serology; and 3) good quality control for genotyping. Our these strengths may help minimize the confounding issues from mixed tumor sites, and serological HPV16 status and significantly increase accuracy of the association in this study. Despite of such several strengths, our study also have several limitations. These include 1) no frequency matching on different demographic, epidemiological, and clinical characteristics between HPV16-positive and HPV16-negative cancer patients in this study; 2) inclusion of small sample sizes and a possible selection bias; 3) potential misclassification of tumor HPV16 status due to limitation of current HPV determination methods; and 4) not possible representation of true prevalence of HPV 16 exposure in the general population when tumor HPV16-negative patients as comparison controls. Thus, prospective, larger or multi-center studies are required to validate our findings in the future.

In conclusion, our present study provides evidence for the first time that three *MDM4* gene polymorphisms could significantly modify individually or in combination the tumor HPV status in SCCOP. *MDM4* genetic variants could be biomarkers for predicting tumor HPV16-positivity of patients with SCCOP, particularly in never-smokers and never-drinkers SCCOP patients. However, validation of these results in larger studies are needed.

## MATERIALS AND METHODS

### Study subjects

A total of 552 incident patients with SCCOP were diagnosed and treated from December 1996 to July 2011 at The University of Texas (U.T.) M. D. Anderson Cancer Center were consecutively recruited. The criteria of inclusion and exclusion were described previously [14]. The study was approved by the Institutional Review Boards of U. T. M.D. Anderson Cancer Center, and all study participants signed informed consent for the sample analysis of genetic variation. All enrolled patients donated 30 ml of blood for genotyping. Paraffin-embedded tumor tissue samples were assessed for HPV16 status. Ever-smokers were defined as patients who had smoked over 100 cigarettes during their lifetimes and otherwise as never-smokers. Drinking status was classified as “ever-drinkers” (alcoholic beverage at least once a week for 1 year) and “never-drinkers” (never had such a drinking pattern).

### MDM4 polymorphism selection/genotyping and HPV16 detection

To select *MDM4* polymorphisms for this study, we used the public HapMap SNP database to identify the tagging SNPs, as we previously reported^19^. All selected SNPs either were directly genotyped or exceeded a threshold level of linkage disequilibrium (LD) value (*r*^2^) with a genotyped SNP within an about 34-kb region of MDM4 gene among a European population. These tagging SNPs were selected according to their pairwise LD with the *r*^2^ threshold of 0.8 and minor allele frequency (MAF) ≥ 0.10. Thus, three tagging SNPs were identified including rs11801299, rs1380576, and rs10900598, in the 34-kb region, and the mean *r*^2^ between the tagging SNPs and their covered but untyped SNPs was 0.98^19^. For the 3 polymorphisms, *MDM4*rs11801299 and *MDM4*rs10900598 are within the 3′ UTR; and *MDM4*rs1380576 is located in the intron 1 of the *MDM4* gene. Genomic DNA before treatment was extracted from blood samples for *MDM4* genotyping. The detail of these methods have been previously described [18, 33]. The paraffin-embedded tissues were used for HPV16 status detection as described previously [5, 24]. For quality control, 10% of samples were retested to verify genotyping and 5% of the tissues samples were retested for tumor HPV16 status. The results of these repeated samples were 100% concordant.

### Statistical analysis

Differences in demographic characteristics, smoking and drinking status, and *MDM4* genotypes between HPV16-positive and HPV16-negative patients were detected using χ^2^ test. Both univariate and multivariable logistic regression analyses were used to estimate the association between *MDM4* genotypes and tumor HPV16 positivity among SCCOP by computing the odds ratios (ORs) and 95% confidence intervals (CIs). The genotype data were further analyzed by stratifying into subgroups by age, sex, ethnicity, smoking and drinking status in multivariable logistic regression models. Statistical Analysis System software (Version 9.3; SAS Institute, Cary, NC) was used to perform all of the statistical analyses. All tests were two-sided, and a *P* value of 0.05 was considered significant.

## Abbreviations

*MDM4*: Mouse double minute 4
HPV: human papillomavirus
SCCOP: squamous cell carcinoma of oropharynx
SNPs: single nucleotide polymorphisms
OR: odds ratio
CI: confidence interval
SCCHN: SCC of head and neck
